# 17-4 Precipitation-Hardening Stainless Steel: Soft-Tough Heat Treatment Mechanism and Thermal Fatigue Characteristics

**DOI:** 10.3390/ma17235851

**Published:** 2024-11-28

**Authors:** Ping-Yu Hsieh, Bo-Ding Wu, Fei-Yi Hung

**Affiliations:** Department of Materials Science and Engineering, National Cheng Kung University, Tainan 70101, Taiwan; n56134273@gs.ncku.edu.tw (P.-Y.H.); n58071065@gs.ncku.edu.tw (B.-D.W.)

**Keywords:** 17-4PH, heat treatment, softening, tensile properties, thermal fatigue

## Abstract

This study selected 17-4PH (Type 630) precipitation-hardening stainless steel as the raw material. After subjecting the material to precipitation hardening and softening heat treatments, the effects of these treatments on the microstructural characteristics and mechanical properties were investigated, along with a thermal fatigue test. The results showed that after the precipitation hardening heat treatment, the ultimate tensile strength and hardness of 17-4PH stainless steel increased. However, the ductility was around 2%, indicating a severe brittleness effect, which is unfavorable for ordnance applications. A double aging treatment for 12 h resulted in a combination of softness and ductility, with an ultimate tensile strength of 900 MPa, ductility of 26%, and hardness HRC 28. These material properties are suitable for cold forging in ordnance and other applications. In addition, a thermal fatigue test was conducted on the softened 17-4PH material from 750 °C to room temperature. After 3000 thermal cycles, there was no significant change in material properties, ensuring it can withstand 3000 rounds of gunfire in a barrel with excellent durability. Furthermore, XRD and fracture surface analysis confirmed that the softening mechanism of 17-4PH stainless steel and its high-temperature phase stability could provide a reference for cold forging and high-temperature applications.

## 1. Introduction

In the AISI classification system, the 6-series stainless steels are known as precipitation-hardening stainless steels. Among these, 630 stainless steel is a martensitic precipitation-hardening stainless steel containing approximately 17 wt.% chromium and 4 wt.% nickel, commonly referred to as 17-4PH stainless steel. 17-4PH stainless steel boasts an impressive combination of high strength, exceptional ductility, ease of workability, and excellent corrosion resistance [1,2,3]. These qualities make it particularly suitable for demanding environments where mechanical performance and durability are paramount. As a result, it finds widespread application in the aerospace and marine industries, where materials must withstand extreme conditions, such as high stresses and exposure to corrosive elements. Its reliable properties make it an ideal choice for manufacturing various precision components, where accuracy and resilience are crucial for optimal performance in critical applications [4,5,6].

The current literature on 17-4PH stainless steel primarily emphasizes the aging hardening heat treatment process due to its widespread application in industries requiring high strength and corrosion resistance. The conventional heat treatment process for 17-4PH stainless steel involves a two-stage procedure: initially, a solution treatment at 1050 °C for 0.5 h, followed by air cooling to room temperature. This first stage forms a martensitic structure with a high dislocation density, contributing to its inherent hardness and strength [7,8]. Subsequently, the material undergoes an aging treatment in the 480–620 °C temperature range. During this aging phase, Cu-rich precipitates form within the martensitic matrix at temperatures exceeding 450 °C; these precipitates are spherical with a diameter of about 2 nm. They are abundant at this stage and exhibit a body-centered cubic (BCC) crystal structure coherently with the matrix [9]. The formation of Cu-rich precipitates significantly enhances hardness through precipitation hardening [10,11,12,13,14]. This hardening effect compensates for the concurrent reduction in dislocation density within the martensitic laths, which would otherwise lead to material softening [15].

During cooling, the martensitic transformation in 17-4PH stainless steel is initiated at approximately 140 °C (Ms point) [7,9]. Upon completion of air cooling to room temperature, the material exhibits a lath-like martensitic structure characterized by high hardness [16,17,18]. While advantageous for many applications, this martensitic microstructure poses challenges for processes requiring high material ductility or reduced hardness. For instance, in the manufacturing of gun barrels, 17-4PH stainless steel must meet stringent mechanical requirements, including hardness of HRC 28~32, to facilitate cold forging [19]. Achieving these properties necessitates a tailored softening heat treatment to modify the martensitic structure without compromising the material’s inherent corrosion resistance or fatigue performance.

While the strengthening characteristics of precipitation-hardening stainless steels have been extensively studied [20,21,22,23], research focusing on their softening treatments remains limited [24]. This gap is particularly evident in studies addressing the specific requirements of cold forging applications. Moreover, the complex thermal fatigue behavior and failure mechanisms of 17-4PH stainless steel in high-temperature, cyclic loading conditions, such as those encountered in gun barrel applications, still need to be fully understood. These aspects are critical for optimizing material performance and extending the service life of components subjected to demanding operational environments.

Given these considerations, there is a growing need for comprehensive research on the softening heat treatment, thermal fatigue properties, and failure mechanisms of 17-4PH stainless steel. Such investigations are vital for advancing its applications in critical industries, particularly those involving dynamic loading and stringent mechanical property requirements.

This study explores the microstructure and mechanical properties of the raw material, furnace-cold material, and precipitation-hardened material. Appropriate heat treatment is applied to develop softening conditions that meet the requirements for cold forging. Additionally, thermal fatigue testing simulating 3000 rounds of gunfire is conducted to compare the microstructure evolution and mechanical properties before and after thermal fatigue. The results aim to establish optimal softening heat treatment conditions and investigate thermal fatigue resistance. The findings will provide valuable references for applying 17-4PH stainless steel in cold-forging rifling processes and help assess the material’s resistance to thermal fatigue from repeated gunfire.

## 2. Materials and Methods

**Figure 1:**
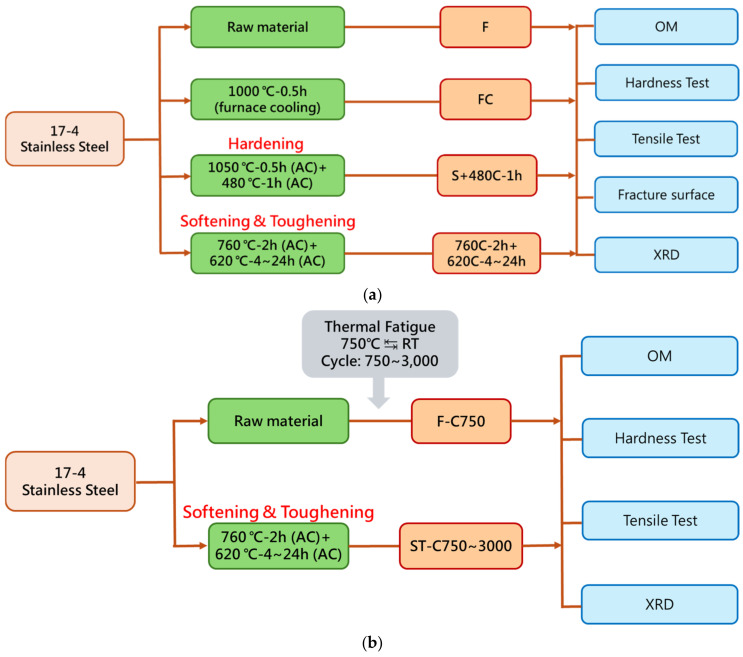
Experimental framework and process flow: (**a**) First two parts; (**b**) third part.

The experimental framework and process flow of this study are shown in Figure 1 and can be divided into three parts:

### 2.1. Investigation of the Raw Material, Furnace-Cooled Material, and Precipitation-Hardened Material [21,22]

**Figure 2:**
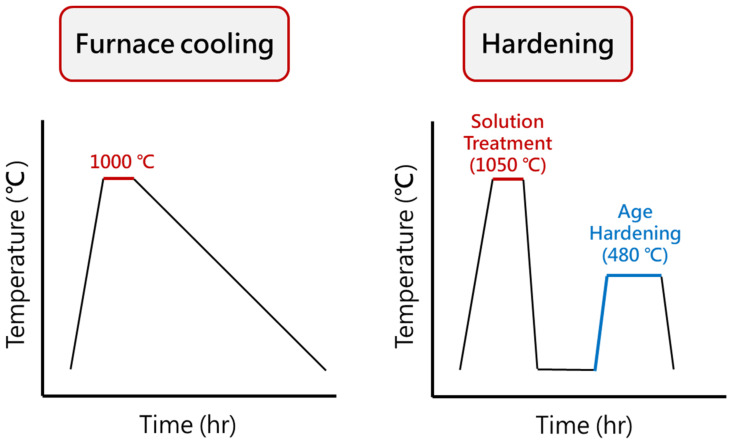
Flowcharts of furnace cooling, solution treatment, and age hardening.

In this study, 17-4PH stainless steel was selected as the raw material, referred to as “F”. The chemical composition of 17-4PH stainless steel is shown in Table 1. The heat treatment methods included furnace cooling, solution treatment, and aging hardening. Furnace cooling involved heating the specimens in a furnace at 1000 °C for 0.5 h and then allowing them to cool down to room temperature within the furnace, naming this material “FC”. The solution treatment involved heating the specimens in a furnace at 1050 °C for 0.5 h, followed by air cooling to room temperature. After the solution treatment, an aging hardening treatment was applied. Aging hardening was performed by heating the specimens in a furnace at 480 °C for 1 h, followed by air cooling to room temperature. After solution treatment and aging, the material was designated as “S+480C-1h”. The flowcharts of furnace cooling, solution treatment, and aging hardening processes are shown in Figure 2.

### 2.2. Investigation of Softening Heat Treatment Conditions

**Figure 3:**
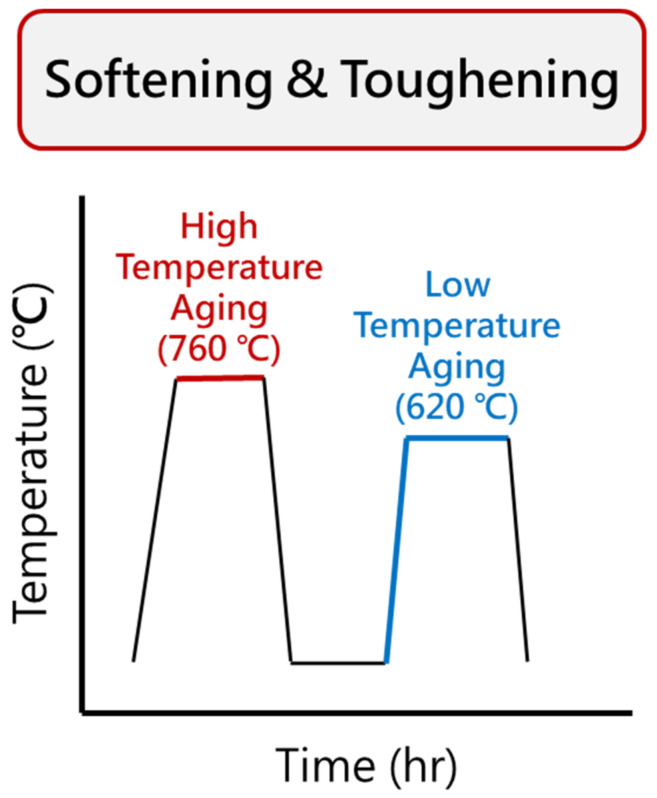
Flowchart of softening heat treatment.

The softening heat treatment in this phase omitted the solution treatment step and directly involved a two-stage aging process [10,24]. The specimens were heated in a furnace at 760 °C for 2 h and then air-cooled to room temperature. Subsequently, the specimens were heated in a furnace at 620 °C for various times (4, 8, 12, and 24 h). The materials were designated as “760C-2h+620C-aging time”, such as 760C-2h+620C-4h, which referred to a specimen that was heat-treated at 760 °C for 2 h, air-cooled, and then heat-treated at 620 °C for 4 h. The softening heat treatment process is illustrated in Figure 3.

### 2.3. Thermal Fatigue Testing of the Raw Material and Soft-Tough Material [25,26]

**Figure 4:**
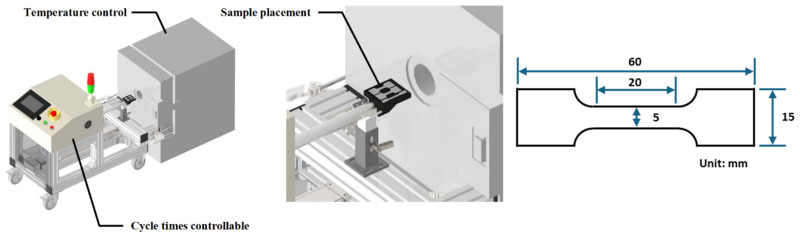
The schematic of the thermal fatigue testing device and the specimens (t = 2 mm).

The raw material (F) and the material with the optimal softening conditions identified in the second phase were subjected to thermal fatigue testing. The thermal fatigue tests were conducted using a thermal fatigue testing machine (TOYO Heat Fatigue CGTH8-L10-400-BR, Toyo, Itami, Japan) under conditions of 750 °C (holding for 30 s) and room temperature (holding for 30 s) in a repeated cycle of 750, 1500, 2250, and 3000 cycles. The material subjected to thermal fatigue was designated as “F-C750” for the raw material and “ST-C750~3000” for the softened material, such as ST-C750 for the softened material after 750 thermal fatigue cycles. ST refers to the soft-tough material from the optimal softening conditions. The schematic of the thermal fatigue testing device is shown in Figure 4.

After polishing, the microstructure of the specimens from the three phases was analyzed using optical microscopy (OM, OLYMPUS BX41M-LED, Tokyo, Japan). Further analysis included Rockwell hardness testing and tensile tests, with the tensile fracture surface observations using scanning electron microscopy (SEM, HITACHI SU-5000, HITACHI, Tokyo, Japan). X-ray diffraction (XRD) analysis was conducted to identify the phase composition in the matrix, with a 2θ range of 30–90 degrees.

## 3. Results and Discussion

### 3.1. Characteristics of the Raw Material, Furnace-Cooled Material, and Precipitation-Hardened Material

**Figure 5:**
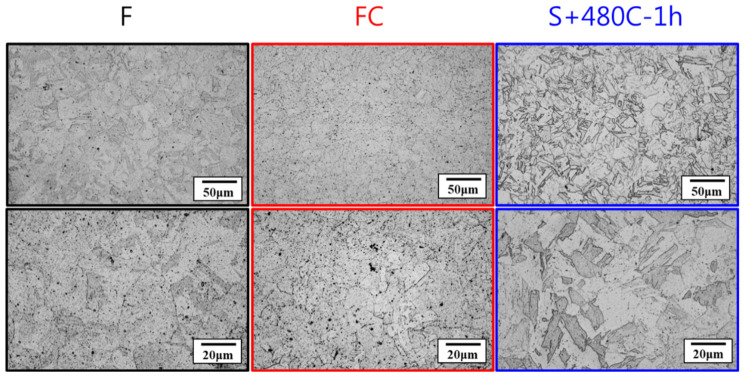
Microstructures of the raw material (F), furnace-cooled material (FC), and precipitation-hardened material (S+480C-1h).

Figure 5 shows the microstructures of the raw material (F), furnace-cooled material (FC), and precipitation-hardened material (S+480C-1h). The precipitation-hardened material exhibited a lath-like martensitic structure compared to the raw and furnace-cooled materials. After low-temperature aging, these martensitic structures displayed stability and strengthening characteristics. Additionally, the precipitation-hardened material contained fine and dense Cu-rich precipitates, contributing to increased martensitic matrix hardness.

**Figure 6:**
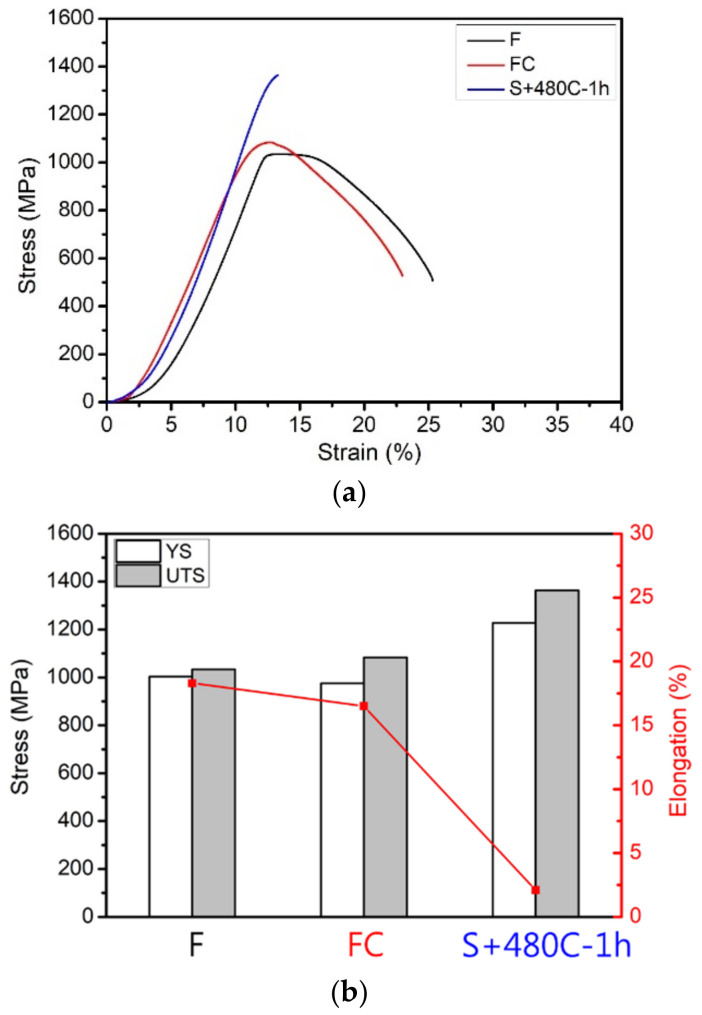

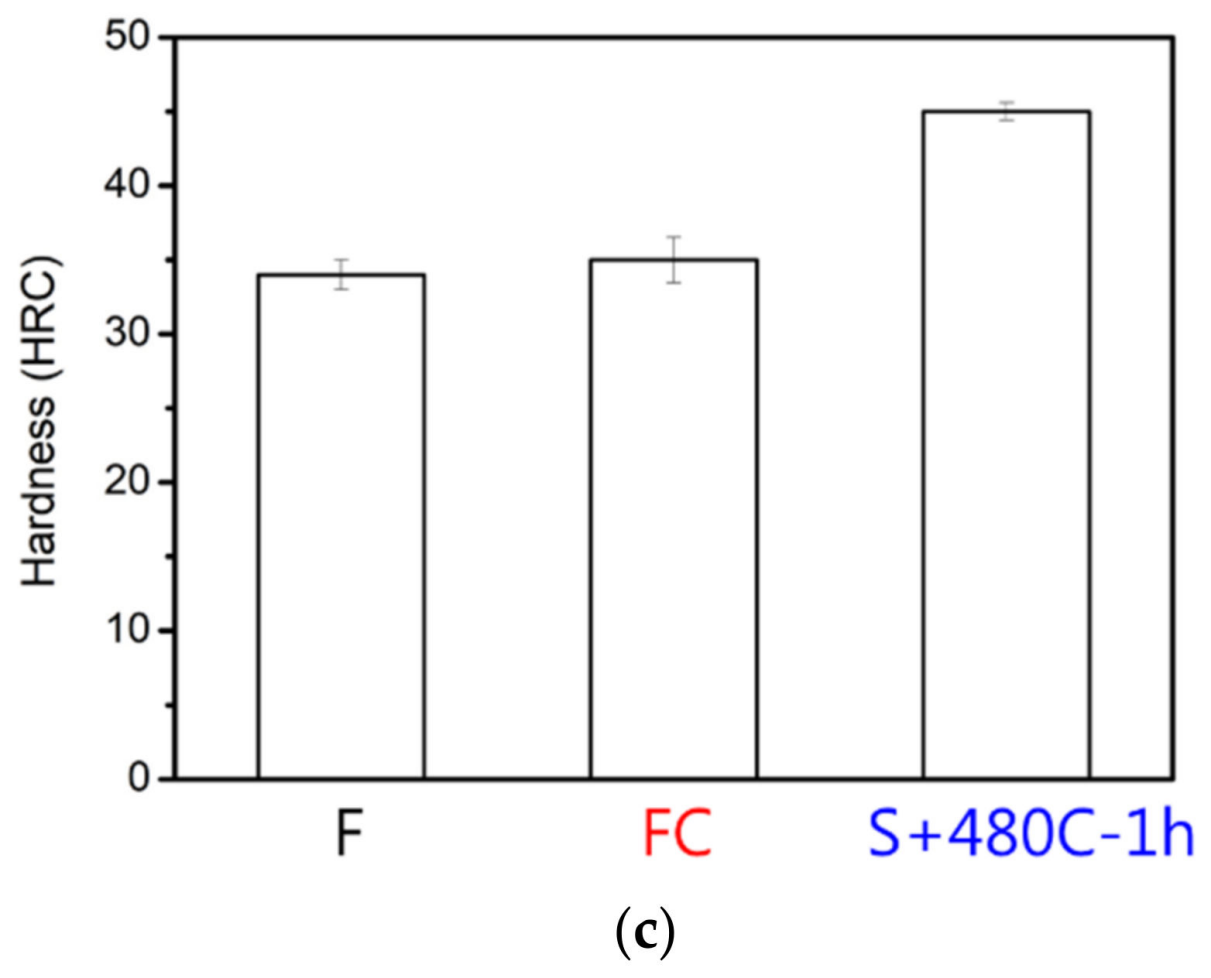
Raw material (F), furnace-cooled material (FC), and precipitation-hardened material (S+480C-1h): (**a**) stress-strain curve; (**b**) YS, UTS, and elongation; (**c**) hardness (HRC).

Figure 6 illustrates the mechanical properties of the raw, furnace-cooled, and precipitation-hardened materials. Figure 6a shows the ultimate tensile properties data, where the tensile strength of the precipitation-hardened material (1364 MPa) was significantly higher than that of the raw and furnace-cooled materials. The factor contributing to strengthening 17-4PH stainless steel is that it transforms into a martensitic matrix after solution treatment. The rapid cooling in the precipitation-hardened heat treatment promotes the formation of the martensitic phase, and precipitation hardening occurs through subsequent aging treatment, further enhancing the strength. Figure 6b highlights the precipitation-hardened material’s excellent yield stress and tensile strength, but its ductility sharply dropped to 2.1%, indicating brittleness that resulted in poor workability. The primary reason for this brittleness is the delicate and uniform precipitation of Cu-rich particles in the martensitic matrix during the aging process, which impedes dislocation movement and substantially increases the hardness of the material [12,13,14]. However, the presence of Cu-rich precipitates also led to a reduction in ductility and an increase in brittleness. On the other hand, these precipitates contributed to the material’s hardness, which reached HRC 45 (Figure 6c).

**Figure 7:**
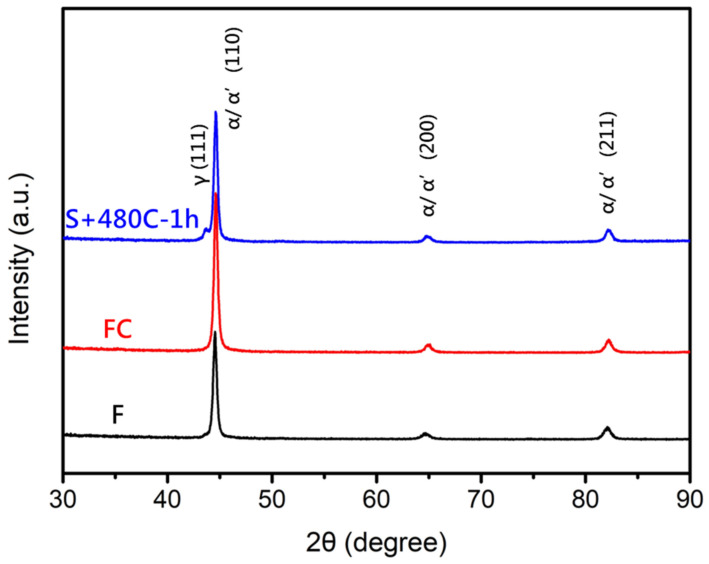
X-ray diffraction pattern of the raw material (F), furnace-cooled material (FC), and precipitation-hardened material (S+480C-1h).

**Figure 8:**
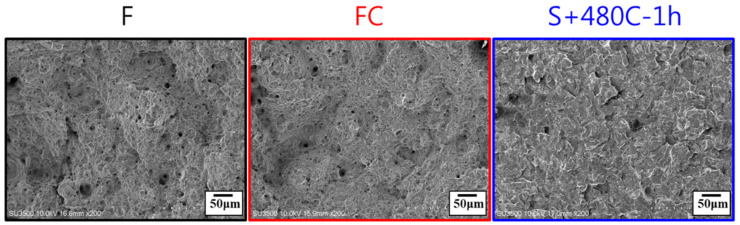
Tensile fracture surface of the raw material (F), furnace-cooled material (FC), and precipitation-hardened material (S+480C-1h).

Furthermore, after high-temperature solution treatment and precipitation hardening, the microstructure of the 17-4PH stainless steel matrix consisted of martensite, ferrite, and retained austenite. Martensite is formed in the material by the rapid cooling of austenite and has higher hardness and strength. Austenite is formed by the dissolution of ferrite in the material at high temperature and is retained during cooling (Figure 7). Tensile fracture surface analysis (Figure 8) revealed that both the raw and furnace-cooled materials retained some ductility, as evidenced by the dimple structures on the fracture surface. In contrast to the raw and furnace-cooled materials, the precipitation-hardened material, with lower ductility, exhibited brittle fracture, characterized by a relatively flat fracture surface, with cracks propagating within the grains, indicating transgranular fracture. Overall, the failure mechanism of precipitation-hardened material is characterized by brittle dominance and poor ductility.

Although the precipitation-hardened material exhibited high strength and hardness, its low ductility was unsuitable for cold forging applications in gun barrel materials. The reason is that cold forging involves significant deformation at room temperature. Materials with low ductility are more prone to cracking or fracturing under such stress because they cannot plastically deform enough without breaking. Gun barrel materials must withstand high forces during manufacturing, and insufficient ductility leads to premature failure. Therefore, this study further investigates the softening heat treatment conditions to adjust the hardness of 17-4PH stainless steel within an appropriate range (HRC 28–30), suitable for cold forging applications.

In this phase, the softening treatment omitted the high-temperature solution treatment step and directly proceeded with a two-stage over-aging heat treatment. The process involved heating the 17-4PH stainless steel at 760 °C for 2 h, followed by air cooling to room temperature. The material was then placed in a furnace at 620 °C for various holding times (4 h, 8 h, 12 h, and 24 h) to investigate the effect of aging duration on material properties.

**Figure 9:**
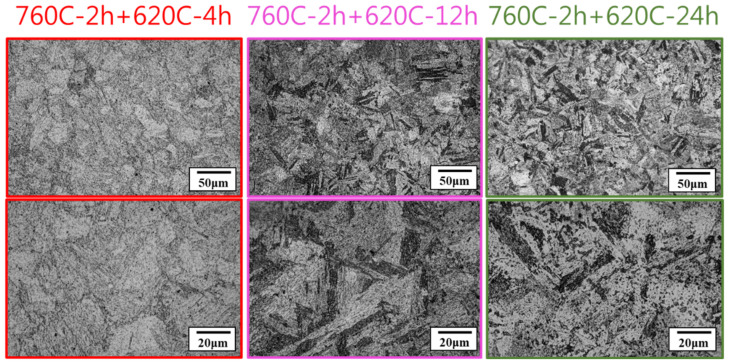
The effect of different aging times on the microstructure of 17-4PH.

Figure 9 shows the microstructures of the soft-tough materials (760C-2h+620C-4h, 12h, 24h). It was observed that after longer aging durations, such as 12 h and 24 h at 620 °C, the lath-like martensitic structures transformed into a layered morphology compared to the shorter duration (4 h). Moreover, after the two-stage aging treatment, the martensitic structure further decomposed, forming fine, needle-like tempered martensite. This type of structure tends to reduce material hardness. Around the Cu-rich precipitates, soft reverted austenite phases may have formed. The two-stage aging process likely increased the content of these reverted austenite phases, further contributing to the reduction in material hardness [10,27]. Based on microstructural data and reference literature, the soft-tough material consisted of tempered martensite, reverted austenite, and coarsened Cu-rich precipitates after the two-stage over-aging heat treatment.

### 3.2. Softening Heat Treatment Conditions and Phase Transformation Mechanism

**Figure 10:**
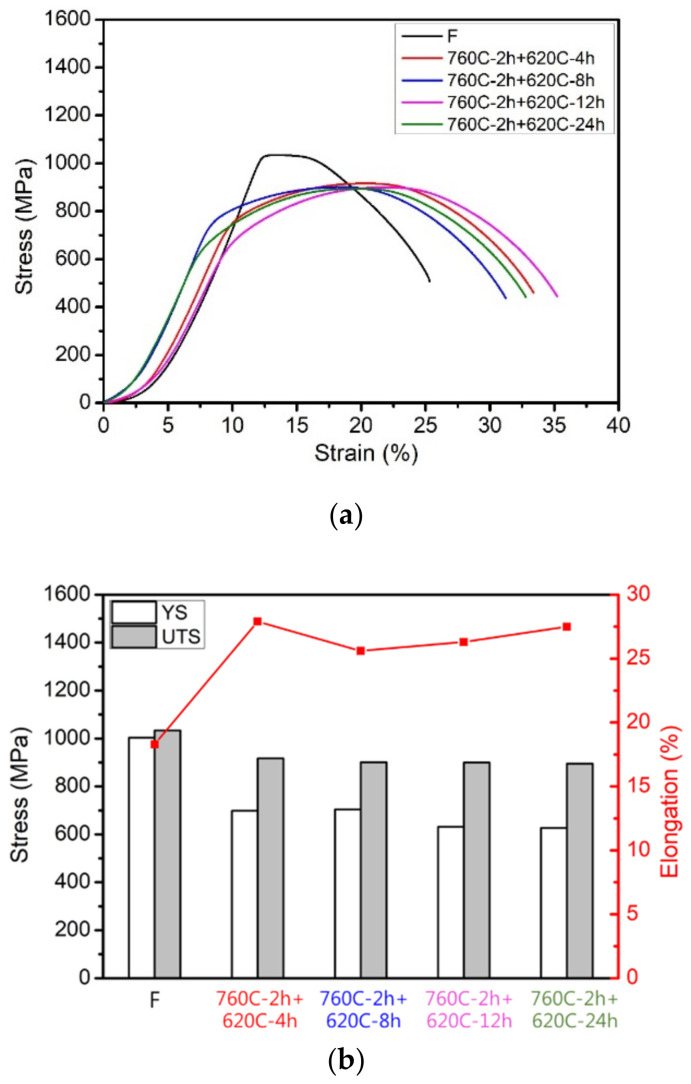

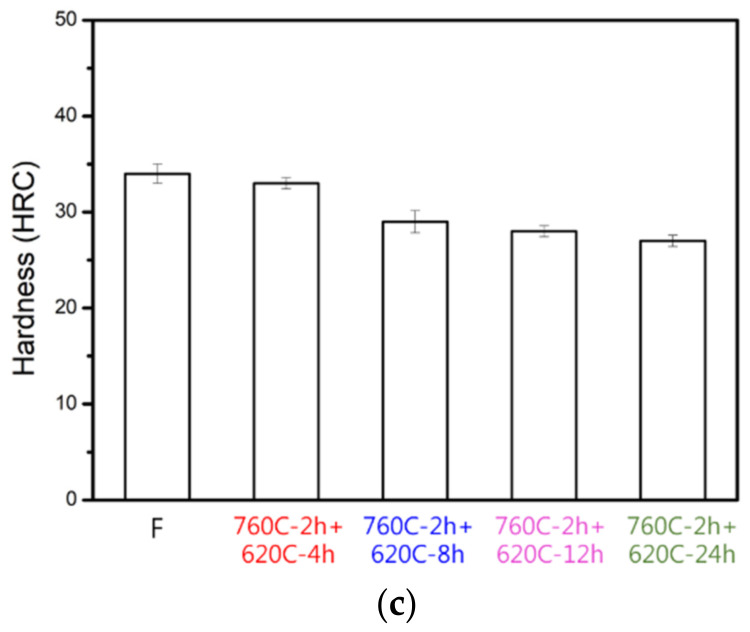
Raw material (F) and soft-tough materials (760C-2h+620C-4h, 8h, 12h, 24h): (**a**) Stress-strain curve; (**b**) YS, UTS, and Elongation; (**c**) Hardness (HRC).

Figure 10 shows the mechanical properties of the soft-tough materials at different aging durations. Figure 10a,b present the tensile properties data. Regardless of the aging duration, the soft-tough materials’ yield stress and ultimate tensile strength were significantly reduced compared to the raw material, while ductility increased to approximately 27%. The fine precipitates formed during earlier aging treatments coarsen significantly. Larger precipitates are less effective at blocking dislocations than fine ones, reducing the material’s ability to resist deformation (hence the drop in yield stress and ultimate tensile strength). The same coarsening of precipitates and reduction in dislocation barriers that reduce strength also allows for more significant plastic deformation. With fewer obstacles to dislocation movement, the material can deform more before fracture, translating into higher ductility. The microstructure becomes less brittle and more capable of absorbing deformation without cracking, increasing ductility to around 27%. Figure 10c shows the hardness data of the soft-tough materials, revealing a clear trend: as the aging duration increased, the material hardness decreased. After 12 and 24 h of aging (760C-2h+620C-12h and 760C-2h+620C-24h), the material hardness was around HRC 28, meeting the requirements for cold forging applications.

**Figure 11:**
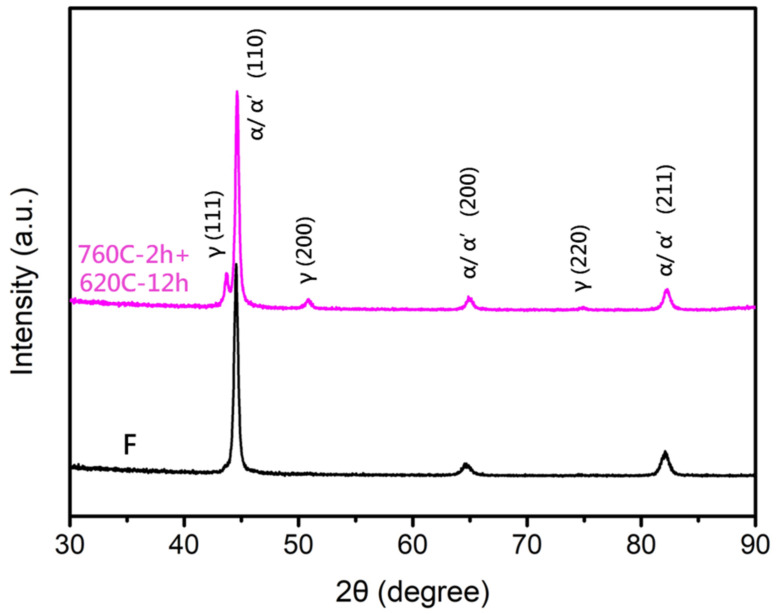
X-ray diffraction pattern of the raw material (F) and soft-tough material (760C-2h+620C-12h).

**Figure 12:**
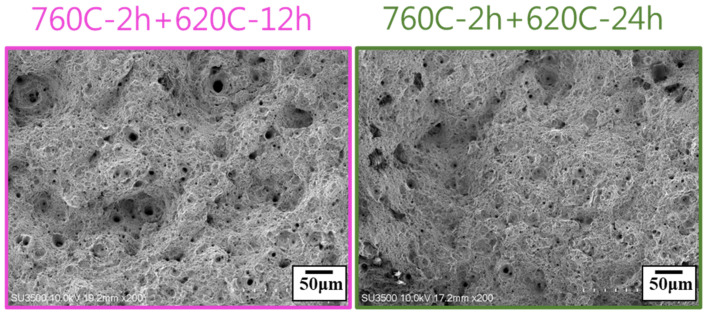
Tensile fracture surface of soft-tough materials (760C-2h+620C-12h, 24h).

XRD analysis of the soft-tough material (760C-2h+620C-12h) is shown in Figure 11, revealing the presence of reverted austenite and martensite in the matrix. The formation of reverted austenite primarily occurs through diffusional reversion, involving the enrichment of austenite-stabilizing elements like nickel and copper. These elements inhibit the transformation of austenite into martensite during cooling. Additionally, as temperature increases, the enrichment of nickel and copper becomes more pronounced, accelerating the nucleation of austenite [24,27]. The tensile fracture surface analysis (Figure 12) showed that, with increasing aging duration (12 h and 24 h), the fracture surfaces exhibited a more significant proportion and size of dimples, corresponding to improved ductility. After the two-stage over-aging heat treatment, the hardness of 17-4PH stainless steel was effectively reduced, and ductility was enhanced, making it suitable for cold forging rifling applications in gun barrels. Considering the heat treatment duration and mechanical properties, it was found that the material treated at 760 °C for 2 h followed by 12 h at 620 °C had an ultimate tensile strength of 900 MPa, ductility of 26.3%, and hardness of HRC 28. These properties met the softening and toughness requirements, so this material was selected for further thermal fatigue testing to evaluate its resistance to the thermal shocks and metallurgical characteristics associated with gunfire.

### 3.3. Thermal Fatigue Failure Characteristics of the Raw Material and Soft-Tough Material

**Figure 13:**
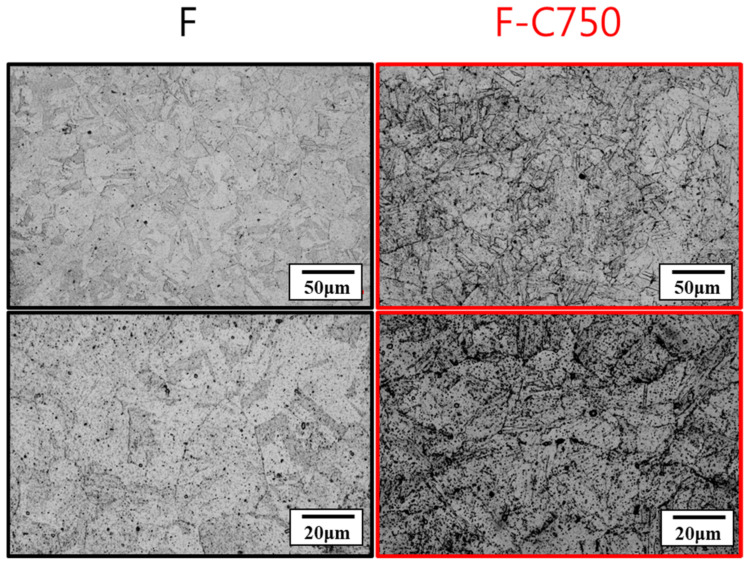
Microstructures of the raw material after 750 thermal fatigue cycles.

**Figure 14:**
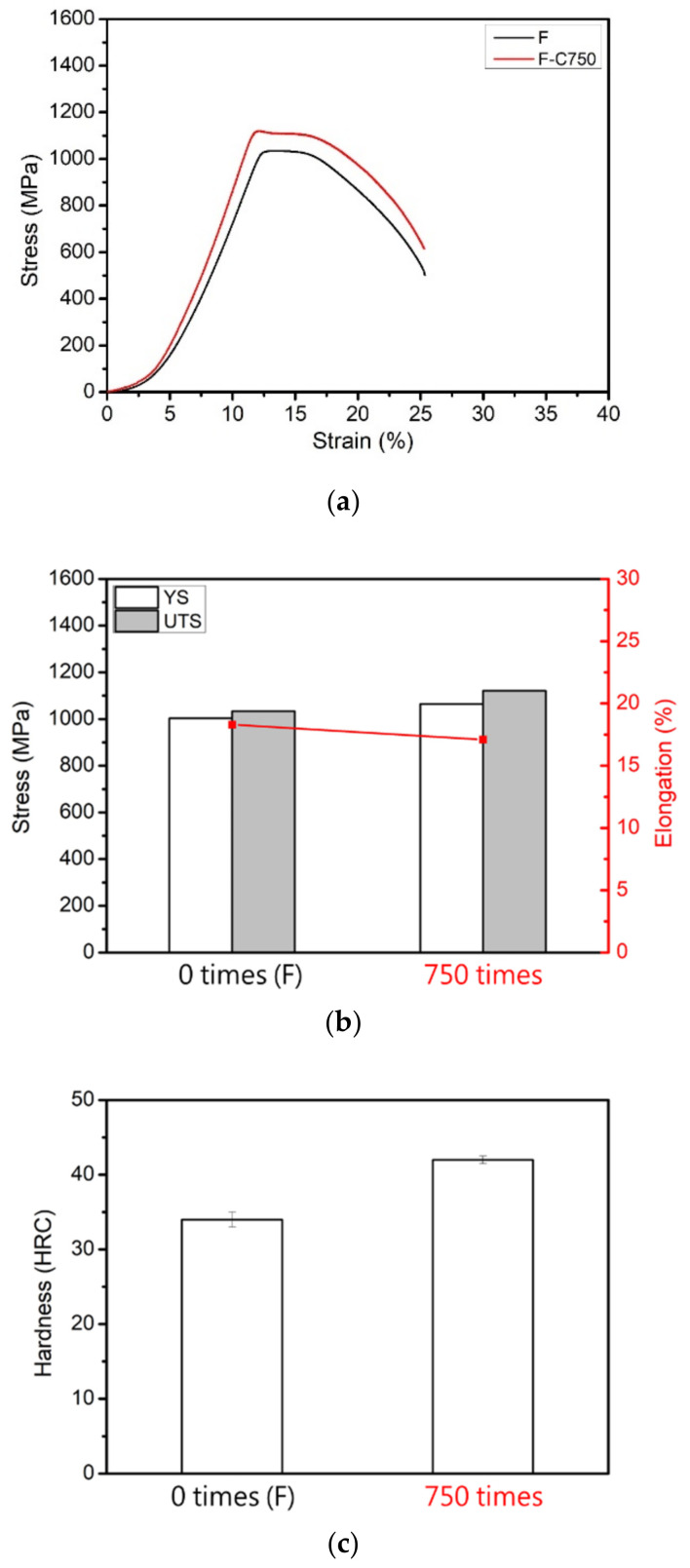
Raw material after 750 thermal fatigue cycles: (**a**) stress-strain curve; (**b**) YS, UTS, and elongation; (**c**) hardness (HRC).

**Figure 15:**
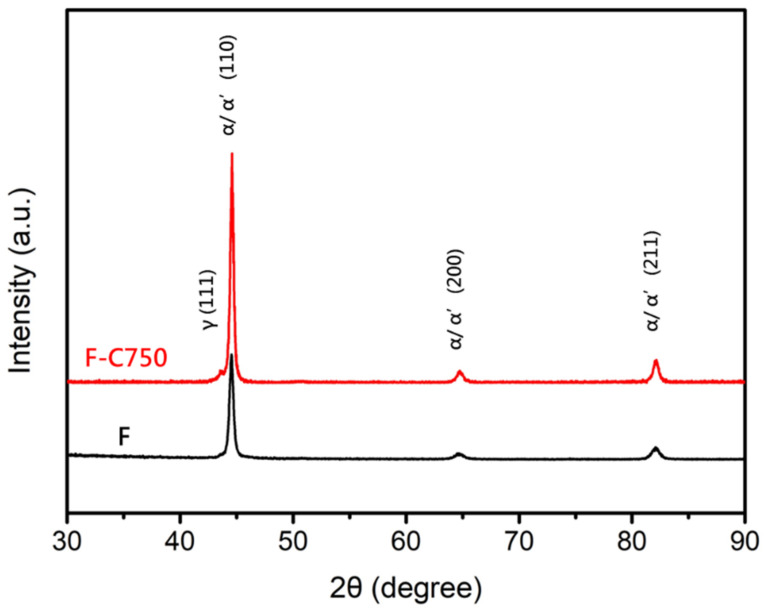
X-ray diffraction pattern of the raw material after 750 thermal fatigue cycles.

In this phase, the effects of thermal fatigue (750 °C ↹ room temperature) on the microstructure and mechanical properties of the raw material (F) and the soft-tough material (760C-2h+620C-12h) were investigated. Figure 13 shows the microstructure of the raw material after 750 thermal fatigue cycles, where refined lath-like martensite structures formed in the matrix. Figure 14 presents thermal fatigue’s effects on the raw material’s mechanical properties. Figure 14a,b show the tensile properties data. After 750 thermal fatigue cycles, the raw material’s yield stress and ultimate tensile strength increased, while ductility decreased slightly. The shear-induced defects from thermal expansion, contraction, and recrystallization behavior drive this grain refinement, resulting in improved tensile properties. Figure 14c shows that the hardness increased dramatically to HRC 42 after thermal fatigue, suggesting that phase transformation occurred, increasing strength and hardness. According to the XRD analysis of the raw material and the material after 750 thermal fatigue cycles (F-C750), changes in the peak intensities of the austenite and martensite phases were observed (Figure 15). The increase in martensite peak and austenite peak intensity contributed to the hardness increase after thermal fatigue. In summary, after undergoing 750 thermal cycles, the strength and hardness of the raw material significantly increased, accompanied by a decrease in ductility. As a result, the raw material does not meet the requirements for high-temperature thermal fatigue resistance.

**Figure 16:**
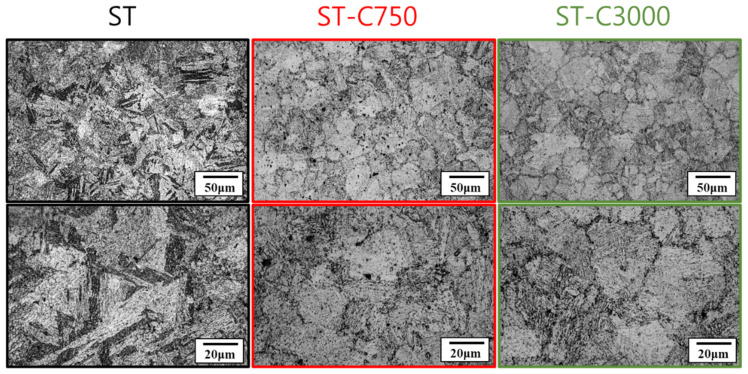
Microstructures of soft-tough material after 750 and 3000 thermal fatigue cycles.

For the soft-tough material (ST), Figure 16 shows that after 750 and 3000 thermal fatigue cycles, the layered martensitic structures in the matrix significantly diminished, and the microstructure became finer. This was primarily due to the repeated thermal expansion and contraction caused by severe temperature fluctuations, which continuously introduced lattice defects and provided nucleation sites for recrystallization [26,28].

**Figure 17:**
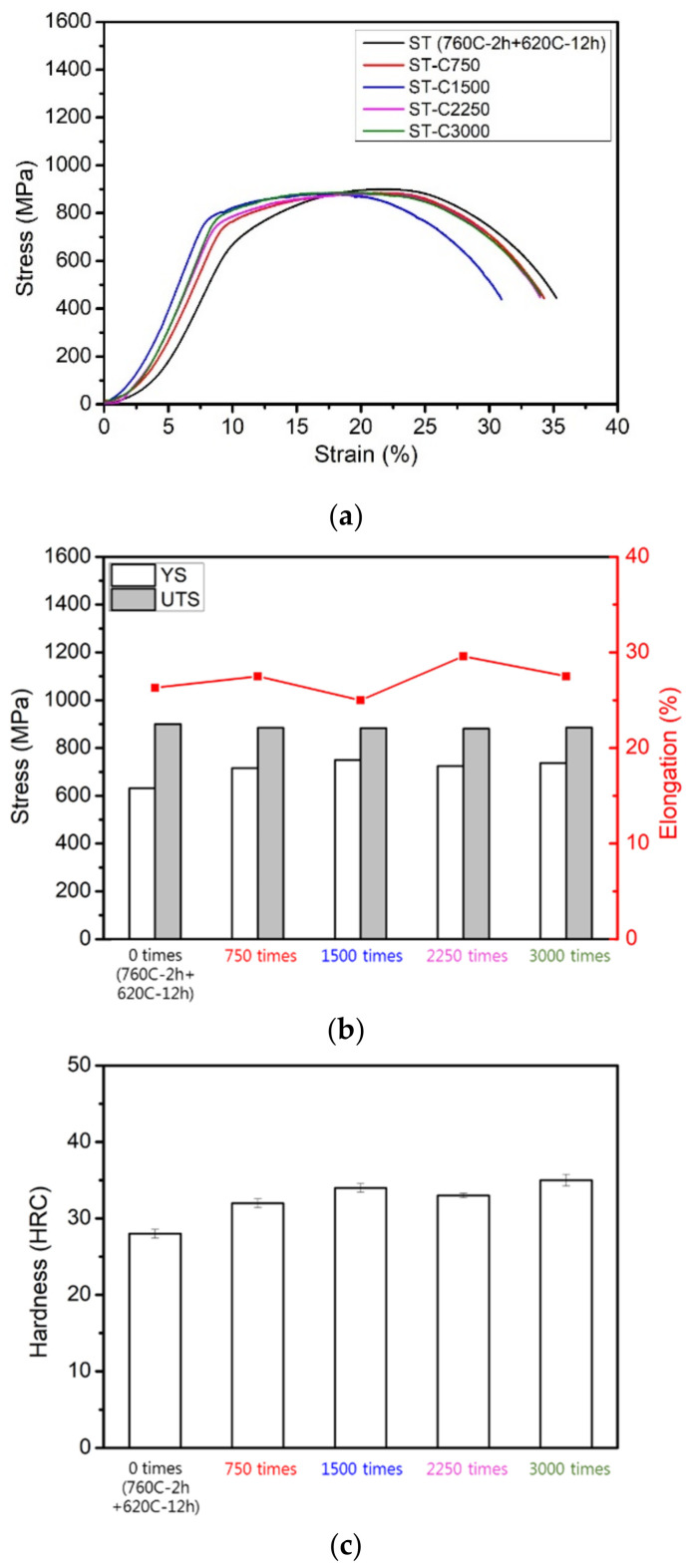
Soft-tough material after 750 to 3000 thermal fatigue cycles: (**a**) stress-strain curve; (**b**) YS, UTS, and elongation; (**c**) hardness (HRC).

**Figure 18:**
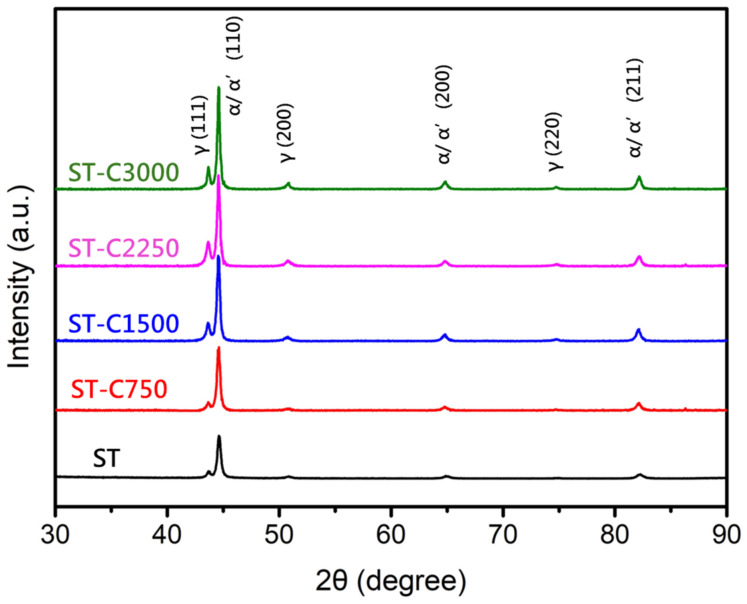
X-ray diffraction pattern of soft-tough material after 750 to 3000 thermal fatigue cycles.

Figure 17a,b show the tensile properties of the soft-tough material after 750 to 3000 thermal fatigue cycles. The yield stress slightly increased, while the ultimate tensile strength and ductility remained close to pre-fatigue levels. After undergoing soft-tough heat treatment, the soft-tough material exhibits fewer stress concentration points and lower residual stress. This means that when the material is subjected to thermal fatigue cycles, the internal thermal expansion and contraction deformations can be relieved through plastic deformation without accumulating excessive stress, thus preventing brittle fracture. Its higher ductility allows the material to adapt effectively to these thermal deformations over multiple cycles. According to the hardness data (Figure 17c), the hardness of the soft-tough material slightly increased to HRC 32 after thermal fatigue. The above results indicate that the softening treatment induces structural transformations and coarsening of precipitates in 17-4PH. These combined effects contribute to the material’s ability to retain its mechanical properties under high-temperature fatigue conditions. However, the material still retained reliability for practical gun barrel applications. Additionally, the XRD diffraction patterns of the soft-tough material before and after thermal fatigue were similar (Figure 18), with only a slight increase in the austenite peak intensity as the number of fatigue cycles increased, indicating no significant phase transformation after thermal fatigue. Therefore, the soft-tough 17-4PH material exhibits excellent high-temperature thermal fatigue resistance, making it a suitable candidate for such applications.

## 4. Conclusions

(1) After conventional solution treatment and subsequent aging hardening of 17-4PH stainless steel, although high strength (ultimate tensile strength of 1364 MPa) and high hardness (HRC 45) were achieved, the material exhibited low ductility (2.1%) with significant brittleness, making it unsuitable for cold forging applications in gun barrel materials.

(2) The softening heat treatment process, which omits the high-temperature solution treatment and directly applies a two-stage aging process, produces a matrix containing reverted austenite and martensite. This softening treatment enables the material to exhibit toughness and ductility, making 17-4PH stainless steel suitable for cold-forging rifling processes in gun barrels.

(3) No significant phase transformation occurred in the soft-tough material after 3000 thermal cycles (750 °C ↹ room temperature). The mechanical properties of the soft-tough material after thermal fatigue testing showed only a slight increase in hardness, with no significant changes in tensile properties. This confirms that the soft-tough material of 17-4PH stainless steel in this study has excellent potential for high-temperature thermal fatigue resistance applications.

## Figures and Tables

**Table 1:** The chemical composition of 17-4PH stainless steel (wt. %).

Element	Cr	Ni	C	Si	Mn
Wt. %	15.00–17.50	3.00–5.00	≤0.07	≤1.00	≤1.00
**Element**	**P**	**S**	**Mo**	**Cu**	**Fe**
Wt. %	≤0.04	≤0.03	≤0.05	3.00–5.00	Bal.

## Data Availability

The original contributions presented in the study are included in the article, further inquiries can be directed to the corresponding author.
